# Inclusive Disaster Relief Lessons learned for People with Disabilities from Hurricane Helene (2024) in North Carolina, USA

**DOI:** 10.1007/s10900-026-01629-z

**Published:** 2026-09-06

**Authors:** Evelyn S. Chiang, Alex Morrison, Alexis S. Cangemi, Justine Richelle, Charlie Marsh, Kristin Shepard

**Affiliations:** 1 Department of Psychology, UNC Asheville, CPO 1630, One University Heights, Asheville, NC 28804 USA; 2 UNC Asheville at the time of this study, Asheville, USA; 3 Department of English, UNC Asheville, Asheville, USA

**Keywords:** Disability, Emergency preparedness, Disaster relief, Developmental disability, Inclusion

## Abstract

People with disabilities are particularly vulnerable to the impact of natural disasters such as hurricanes and tropical storms. They also experience greater difficulty obtaining relief aid as well as recovering from storm-related impacts. The purpose of this study was to examine the experiences of people with disabilities as well as parents of someone disabled following Tropical Storm Helene, which struck the western North Carolina region in late September 2024. In the present study, 40 disabled adults and 28 parents of a disabled child, teen, or adult reported on their experiences with disaster response services following Tropical Storm Helene. Respondents completed surveys consisting of both open-ended and selected-response items. Respondents shared personal experiences with evacuating, accessing community care stations, and barriers to obtaining resources. A number of recommendations are offered to promote more inclusive responses to future natural disasters. These recommendations include clearer communication about the availability of resources as well as providing additional ways to access them.

## Introduction

People with disabilities are particularly vulnerable to disaster due to limited access to essential information, adequate support, and necessary services. Globally, people with disabilities are frequently among the most economically and socially marginalized populations, oftentimes experiencing disproportionately high rates of poverty which can in turn reduce access to health care including routine medical care, rehabilitative services, or assistive devices [1]. Further, people with disabilities tend to have less access to reliable transportation [2], exacerbating their risk when a natural disaster occurs and evacuation is needed. Even mortality rates are higher during disasters for people with physical disabilities, limited mobility, or mental disorders [3]. They are also at higher risk for injury [4], loss of property [5], difficulties during sheltering [6], and more vulnerabilities after the disaster [7]. People with disabilities are often overlooked in disaster relief and the last to receive essential resources [8–9]. Consequently, people with disabilities are often not considered in disaster preparation, leading to limited access to information regarding disasters and unequal access to aid and resources in the aftermath of disasters [10].

It is essential for people with disabilities to be involved in efforts to better plan for and respond to natural disasters. In the immediate aftermath of a devastating event such as a hurricane or tropical storm, impacted residents are in dire need of essential resources such as food, water, gas, and power sources. As relief begins to arrive in impacted areas, the focus is often on making resources available to the community as quickly as possible. For people with disabilities, the presence of resources may not equate to being able to access them.

The purpose of the current study was to examine the impact of Tropical Storm Helene on people with disabilities who were residing in western North Carolina when the storm hit the region in late September 2024. It is hoped that this study will shed greater light on the challenges faced by people with disabilities following natural disasters, as well as inform practices on how to improve accessibility in future disaster response.

## Hurricane/Tropical Storm Helene

On September 27, 2024, Tropical Storm Helene hit the western North Carolina region with devastating impact. After making landfall in Florida as a category 4 hurricane, the center of the storm moved inland across Georgia, South Carolina, and North Carolina where it weakened into a tropical storm as hurricane-force wind gusts continued to impact a wide area [11]. Helene brought 20 to 30 inches of rain to the western North Carolina mountains [11], leading to numerous flash flood emergencies and over 2,200 landslides in the region [12]. In Buncombe County, the French Broad River and Swannanoa River surpassed record flood levels previously set in 1916 [11].

## Impact on Infrastructure

In the immediate aftermath of Helene, residents of western North Carolina lost power, water, cell signal, and access to Internet services. The high winds and large amount of rainfall brought by Helene caused extensive tree damage as well as flooding.

In western North Carolina, more than a million residents lost power [13]. About half of those customers had power restored within a week. However, two weeks after Helene, 25,000 Asheville customers remained without power [11].

The city of Asheville’s water supply sustained severe damage from Helene. The city of Asheville restored non-potable water to residents in the middle of October [14]. This was a critical step in recovery, as it meant residents could once again flush their toilets and boil non-potable water for household use. The city’s boil water advisory was finally lifted 53 days after Helene [11].

In addition to loss of power and water, residents of western North Carolina struggled with communications and accessing information in the wake of the storm. In Buncombe County, reports from the Federal Communications Commission [15] indicated that over 80% of cell sites were out on September 28, the day after Helene passed through the area. Cell sites were gradually restored in the weeks that followed, with 40% of sites remaining out on October 4 (one week after Helene).

Area residents also lost access to Internet services following Helene. Spectrum, one major provider in western North Carolina, reported that 236,000 customers lost services [16]. Even as power was restored, many residents continued to experience difficulty with accessing Internet services.

## Difficulty with Transportation

Flooding, mudslides, fallen trees, downed power lines, and lack of traffic signals created dangerous road conditions in the immediate aftermath of Helene. On September 28th, Buncombe County officials stated that all roads in Western North Carolina were closed [17].

North Carolina’s Department of Transportation [18] found 6,900 sites where roads and bridges were damaged. Accessibility was so challenging that helicopters were needed to rescue people in Chimney Rock and Lake Lure [17], and volunteers used mules to bring food and supplies to some areas [19].

Public transportation was unavailable for weeks following Helene. Asheville city bus (ART–Asheville Rides Transit) began operating again on October 7, serving select routes with modified schedules that ended at 5pm [20]. Services gradually increased in the weeks that followed [21].

## Community Care Stations

In the aftermath of Hurricane Helene, Buncombe County established eight community care stations to address the immediate and ongoing needs of those affected by the storm. These stations served as centralized hubs where community members could access essential resources such as picking up packaged water, refilling containers of water, picking up food items, showering, utilizing laundry facilities, charging phones, accessing the Internet, and seeking basic medical assistance. Spread throughout the county, community care stations were located in shopping plazas, schools, churches, or other locations accessible by the community.

## Methods

### Procedures

The present study was approved by the Institutional Review Board (IRB) at the institution of the first author [2340864-1]. The study was conducted in July 2025, about 10 months after Helene hit the region. It was open to any individual with a disability as well as any parent of a person with a disability who was residing in western North Carolina when Helene struck the region in September 2024. Participants were recruited via social media, digital announcements, flyers, and word-of-mouth.

Using Qualtrics, the researchers obtained the informed consent of all participants by providing information about the study and requesting that respondents affirm their consent to participate prior to beginning the study. Respondents then completed the questionnaire which consisted of both selected-response and open-ended items. Participants were entered into a drawing for one of three $25 gift cards and otherwise not compensated for participation.

### Participants

A total of 40 adults with disabilities and 28 parents of a disabled child, teen, or adult participated in this study. Among the disabled adults, age ranged from 18 to 72 (median age 36). In terms of gender, 25 identified as female, six as male, and nine as agender, gender-queer, gender non-conforming, non-binary, or transgender.

With regard to disabilities, the majority of disabled adults indicated being autistic (*n* = 25); 14 reported having ADHD, 17 a mental health condition, and 13 a chronic illness. Respondents also reported having an intellectual or cognitive disability, learning disability, vision impairment, mobility impairment, seizure disorder, traumatic brain injury (TBI), or hearing impairment. Twenty-nine respondents indicated having more than one disability.

Among the parents of someone disabled, age ranged from 30 to 73 (median age 47.5). Their children ranged in age from 1 to 52 (median age 12). In terms of gender of the parents, 27 identified as female and one as non-binary. Among their children, seven were female, 15 were male, and six were non-binary or transgender.

The majority of parents indicated that their child was autistic (*n* = 24); 17 reported their child had ADHD, eight had cerebral palsy, and seven had a mental health condition. Respondents also reported that their child had an intellectual or cognitive disability, learning disability, vision impairment, mobility impairment, seizure disorder, chronic illness, or TBI. Twenty-three parents indicated their child had more than one disability. Table 1 displays the reported disabilities of both the disabled adult respondents and the children as indicated by their parents.

**Table 1:** Reported Disabilities of Adults and Children (*N* = 68)

Disability	Number of Adults^a^	% of Adults	Number of Children^b^	% of Children
Attention-Deficit/ Hyperactivity Disorder (ADHD)	14	35.00%	17	60.71%
Autism	25	62.50%	24	85.71%
Intellectual or Cognitive Disability	3	7.50%	5	17.86%
Learning Disability	5	12.50%	5	17.86%
Vision Impairment	6	15.00%	1	3.57%
Cerebral Palsy	0	0%	8	28.57%
Mobility Impairment	8	20.00%	4	14.29%
Mental Health Condition	17	42.50%	7	25.00%
Seizure Disorder	5	12.50%	3	10.71%
Chronic Illness	13	32.50%	3	10.71%
Traumatic Brain Injury	2	5.00%	1	3.57%
Hearing Impairment	2	5.00%	0	0%

Individuals were able to indicate multiple disabilities. Nine adults indicated having a disability other than those listed. Three parents indicated that their child had an additional disability aside from those listed.

^a^*n* = 40

^b^*n* = 28; children’s disabilities were reported by their parents

## Results

The online questionnaire consisted of several sections asking respondents about their experiences with evacuating the region after the storm, utilizing community care stations, and barriers to accessing resources. Items consisted of both selected-response and open-ended items. Respondents were assured that their survey responses would be kept confidential and were able to skip questions or cease participation at any time without penalty. The results of quantitative items are reported through frequency analysis, and selected open-ended responses are shared in the results below.

### Evacuating after Helene

On September 30, three days after Helene passed through western North Carolina, the population of Buncombe County (which includes the city of Asheville) was at -56% below baseline, with the city itself at -52% below baseline. By October 5, Asheville’s population was at 72% of its pre-storm numbers [22]. Though evacuation length varied from household to household, these numbers provide a sense of the significant proportion of residents who fled the area.

Disabled individuals and households with disabled family members are more likely to experience difficulty evacuating from a natural disaster. In the present study, respondents were asked if they experienced difficulty evacuating their homes. Among the disabled adults in the sample, 22 reported that they did not experience difficulties, 18 of which did not evacuate at any time after Helene. Of the 18 respondents that did experience difficulty evacuating, 12 were able to evacuate and six were not. Thirteen respondents detailed the length of their evacuation: less than a week (*n* = 2), less than a month (*n* = 4), one to two months (*n* = 3), and two months or more (*n* = 4).

From a menu of selected-response items listing possible reasons for evacuating, displayed in Table 2, the most common reason cited for evacuation was lack of power (*n* = 18), closely followed by lack of water (*n* = 17), reliable cell service (*n* = 17), and reliable Internet service (*n* = 15). Respondents also indicated evacuating due to lack of potable water specifically, the inability to acquire resources for survival, damage to the residence that made it unsafe, and difficulty accessing the home.

**Table 2:** Reported Reasons for Evacuation (*N* = 39)

Reason	Number of Disabled Adults^a^	% of Disabled Adults	Number of Parents^b^	% of Parents
Lack of power	18	81.82%	15	88.24%
Lack of water	17	77.27%	14	82.35%
Lack of potable water	13	59.09%	12	70.59%
Lack of reliable cell service	17	77.27%	14	82.35%
Lack of reliable Internet service	15	68.18%	13	76.47%
Inability to acquire resources for survival (such as food, water)	13	59.09%	11	64.71%
Damage to the residence that made it unsafe	4	18.18%	2	11.76%
Difficulty accessing the home (e.g., trees blocking the road or driveway)	4	18.18%	3	17.65%

Individuals were able to indicate multiple reasons for evacuating. Three adults indicated a reason for evacuating other than those listed. Five parents indicated a reason for evacuating aside from those listed.

^a^*n* = 22

^b^*n* = 17

More information on reasons for evacuation was collected via an open-ended response. In describing difficulty accessing their residence, one respondent noted, “we had a mud slide and the parking lot was full of mud, so my home was accessible but it was harder to get to and much more difficult/unsafe.” Several disabled adults detailed specific issues related to healthcare that led them to evacuate. For example, one cited needing to evacuate due to “Lack of access to regular medical care due to prolonged office closures.” Another respondent noted a lack of support, stating, “My caregivers were also displaced and arranging resources for returning. I had to rely on other family members to care for me.”

Among the parents of a disabled person, 18 parents reported that they did not experience difficulties evacuating; of these, 12 did not evacuate for any time period following Helene. Eight respondents reported difficulty evacuating; of these, seven eventually were able to evacuate whereas one was not. Twelve parents provided information on the length of their evacuation: less than a week (*n* = 2), about a week (*n* = 5), 10 days (*n* = 1), three weeks (*n* = 2), and a month (*n* = 2).

In this group, the most common reason for evacuation was lack of power (*n* = 15); this was closely followed by lack of water (*n* = 14), reliable cell service (*n* = 14), and reliable Internet service (*n* = 13). Respondents also cited reasons such as lack of potable water, inability to acquire resources for survival, damage to the residence that made it unsafe, and difficulty accessing the home.

One parent described evacuating after experiencing the immense difficulty of the storm’s immediate wake: “the trauma of the four days without access to basic necessities was too much and I needed support while I processed and started to heal.” Several parents indicated that they experienced many of the difficulties listed but ultimately remained at their residence. One explained, “We had no power, no water, Limited cell, limited Internet, etc. but were unable to evacuate due to caregiving needs.”

### Accessing Community Care Stations

Over the course of Helene recovery, Buncombe County set up eight separate community care stations that provided a number of resources and services. The first station opened on October 10, 2024 [23], and the last one closed in May 2025 [24]. Additional sites offering resources were provided by private entities such as banks and churches.

In the present study, thirty-two of the disabled adults in the sample provided information on the resources and services they utilized from these stations. The most common resource acquired was packaged water bottles or boxes of water (*n* = 26), followed by pre-packaged food (*n* = 22). Respondents also visited these stations to sign up for FEMA assistance (*n* = 19), to eat or pick up hot meals (*n* = 18), and to refill water containers (*n* = 17). Additionally, respondents used shower trailers, medical services, and mobile laundry services. Table 3 displays respondents’ cited use of community care stations.

**Table 3:** Resources and Services Utilized at Community Care Stations (*N* = 52)

Resource	Number of Adults^a^	% of Adults	Number of Parents^a^	% of Parents
Picking up pre-packaged water bottles or boxes of water	26	81.25%	17	85.00%
Refilling your own containers of water	17	53.13%	13	65.00%
Picking up pre-packaged food (such as cans, boxes, MREs, etc.)	22	68.75%	17	85.00%
Eating or picking up hot meals	18	56.25%	12	60.00%
Shower trailers	7	21.88%	5	25.00%
Mobile laundry facilities	5	15.63%	7	35.00%
Medical services	6	18.75%	1	5.00%
Signing up for FEMA assistance	19	59.38%	11	55.00%

Individuals were able to indicate multiple reasons for accessing the community care centers. Nine disabled adults indicated reasons other than those listed. Four parents indicated resources aside from those listed.

^a^*n* = 32

^b^*n* = 20

Respondents indicated barriers for utilizing resources at community care stations by selecting from a list of potential barriers; these are displayed in Table 4. The most common barrier was lacking basic operating information (*n* = 16). Respondents also reported difficulties due to sensory overwhelm (*n* = 14), an inability to wait in long lines or crowded spaces due to health or disability (*n* = 14), and lacking access to the Internet, a working phone, or technology to get information about these stations (*n* = 14).

**Table 4:** Reported Barriers to Accessing Resources at Community Care Stations (*N* = 47)

Barrier	Number of Adults^a^	% of Adults	Number of Parents^b^	% of Parents
I did not know these resources were available.	13	38.24%	2	15.38%
I did not know basic operating information about these resources, such as location, hours of operation, etc.	16	47.06%	3	23.08%
I did not have adequate information on how to use the resources, such as how the mobile laundry facilities worked.	8	23.53%	1	7.69%
I could not access the resources due to lack of transportation.	7	20.59%	2	15.38%
I could not physically access the resources due to lack of accessibility. For example, the laundry trailers or showers were inaccessible.	4	11.76%	1	7.69%
I had difficulty communicating with the people working at these facilities.	3	8.82%	1	7.69%
I had concerns about interacting with the people working at these facilities.	9	26.47%	2	15.38%
I had concerns about interacting with people using these facilities.	3	8.82%	1	7.69%
I was concerned about my privacy or confidentiality at the resource site.	5	14.71%	0	0%
I/my family felt unwelcome or unsafe at the facility.	4	11.76%	1	7.69%
I was not sure if I/we qualified for the resource.	7	20.59%	10	76.92%
The staff or volunteers were not trained in disability, trauma, or cultural sensitivity.	5	14.71%	1	7.69%
I/we could not access the resource because of sensory overwhelm (noise, crowds, lights, etc.).	14	41.18%	3	23.08%
I/my child was unable to wait in long lines or crowded spaces due to my health or disability.	14	41.18%	1	7.69%
I needed a support person or interpreter and none were available.	3	8.82%	0	0%
I was not able to leave my home due to caregiving responsibilities.	0	0%	4	30.77%
I did not have access to the internet, a working phone, or technology to get information.	14	41.18%	7	53.85%
I had limited English proficiency and materials were not available in my language.	0	0%	0	0%
I had limited literacy and information was not provided in plain or easy-to-understand language.	0	0%	0	0%
I did not trust the organization running the resource site.	3	8.82%	0	0%
I feared involvement from government or law enforcement agencies.	4	11.76%	1	7.69%
I had past negative experiences at similar resource sites.	1	2.94%	0	0%
I did not have the energy or capacity to travel or advocate for myself.	13	38.24%	3	23.08%

Individuals were able to indicate multiple barriers to accessing resources they experienced.

^a^*n* = 34

^b^*n* = 13

In an open-ended response, 27 participants offered suggestions for improvement. Some suggestions focused on how the staffers at these sites acknowledge and support those with disabilities. One respondent recommended having “Some sort of disability-trained assistant at the FEMA facility,” an individual who could “interpret for them,” as well as “Disability guides at stations in a particular color of vest that were available to help.” Other recommendations spoke to the difficulty getting to community care stations given difficulty with travel. In terms of offered solutions, one participant suggested “an option for delivery or curbside pickup.” Another explained, “It would have been lovely if FEMA had worked with another organization (Meals on Wheels perhaps) to deliver resources, instead of insisting folks came to them, when limitations to transportation is not uncommon.”

In the sample of parents of someone disabled, the majority of respondents reported visiting these stations to pick up packaged water bottles or boxes of water (*n* = 17) or pre-packaged food (*n* = 17). Additionally, parents visited community care stations to refill containers of water (*n* = 13), to eat or pick up hot meals (*n* = 12), and to sign up for FEMA services (*n* = 11). Respondents also utilized the shower trailers, mobile laundry facilities, and medical services at these stations.

Thirteen parents reported experiencing barriers when accessing resources at community care stations. The most commonly cited barrier was not being sure if they qualified for the resource (*n* = 10). Many parents indicated that they did not have access to the Internet, a working phone, or technology to get information about these stations (*n* = 7). Additional barriers included the inability to leave the home due to caregiving responsibilities, insufficient energy or capabilities to travel or advocate for oneself, and sensory overwhelm.

In an open-ended response about how access to these resources could be improved, parents offered a variety of suggestions. Two parents cited the difficulty with showering, with one parent suggesting family shower stations, and another describing the inaccessibility of shower and laundry trailers at a particular site: “all the laundry [and shower] trailers at the Candler site had stairs leading up to them. This would have been difficult for anyone (including my son) with a severe mobility disorder to access, as the stairs were small and had no handrail.”

### Difficulty Accessing Resources

Although community care stations provided essential resources for survival, including food and clean water, many western North Carolina residents struggled to obtain basic resources.

When asked if there were any resources they needed but could not access in an open-ended response, the most common responses from disabled adults were food, water, and medical supplies/services. In terms of food, one adult reported dietary restrictions severely limiting their options, while another shared, “We did not have access to pre-prepared or hot meals that I did not still have to cook.” Additionally, one respondent that struggled with water and proper medical care wrote, “I did not have a way to store medications, or immediate access to water I could drink.” The second most common responses were struggles with laundry trailers and financial assistance.

The parents in the sample reported difficulties accessing gas and food. One respondent shared, “I needed specific foods for my daughters that were hard to find when we didn’t have access to money for stores.” This was a particular issue for individuals with dietary restrictions, food sensitivities, or food aversions. Additionally, one respondent emphasized the need for ongoing care, citing the need for “Resources to help support my family during these hard times even after several months later.” The recovery process for many extended far beyond the weeks immediately following Helene.

Residents of western North Carolina who were impacted by Helene could apply for federal disaster assistance. There were three ways to apply for FEMA assistance: by filling out information on a website, by phone, or by downloading the FEMA app [25]. In the immediate aftermath of the storm, many residents were unable to pursue any of these avenues given the lack of cell signal and Internet access. Eventually, there were physical locations set up to help residents apply for FEMA assistance. In addition to challenges with the application process, residents experienced difficulties with verifying damage to residences and receiving aid.

Both disabled adults and parents of someone disabled reported difficulty with getting aid through FEMA. Five of the disabled adults described difficulty accessing FEMA services. One respondent detailed, “I attempted to access FEMA support services through phone, online, and in-person. It was a difficult and confusing process.” Another described “long waits, poor organization, etc.”

## Discussion

Natural disasters have significant and varying impacts on the residents in an affected area. In western North Carolina, the natural land topography consisting of mountains, rivers, and creeks led to substantial differences in Helene’s impact: Some areas experienced immense damage due to flooding, landslides, and fallen trees, while other places had minimal to no damage. The capacity to respond to a disaster can also vary significantly from household to household. Pre-storm financial resources play a large role in the ability to evacuate, secure necessities for survival in the immediate aftermath, and recover from the disaster.

People with disabilities experience higher rates of poverty compared to their nondisabled counterparts. Lack of adequate financial resources likely contributed to the difficulties and inability to evacuate following Helene. Nearly half of the disabled adult respondents reported difficulty evacuating, with a third of these individuals ultimately unable to evacuate. Given that many residents in the area did not have power for at least a week or two, and that the city of Asheville was without potable water for over seven weeks, the inability to evacuate meant significant hardship for everyday survival.

Following Helene, Buncombe County set up eight community care stations across the area. A number of non-profits and other entities set up stations as well. However, comprehensive information listing every available station, with basic operating information such as location, hours, accessibility, and the resources and services provided, was not available.

Given the potential difficulties with communication in the immediate aftermath of a storm, it is important for officials to provide essential information in advance of an impending natural disaster. Cities and counties might identify potential locations for informational hubs that are intended to serve the public following the event. In addition, official channels can provide information about resources that are specifically designed to support to disabled residents. For instance, the Disability & Disaster Hotline is available 24/7 and provides a variety of resources including urgent response referrals [26].

The availability of resources is only the first step in disseminating resources. Some disabled residents may not be able to access resources available at community care stations. For instance, many respondents reported difficulties with sensory overwhelm and waiting in long lines. To increase accessibility, these stations might offer clear signage detailing resources, procedures for obtaining them, as well as the designation of a staff person who can assist disabled patrons. Having the option for “curbside delivery” of resources could also be helpful for many. For residents unable to leave their homes or otherwise travel to these stations, it would be important to organize staffers or volunteers who can deliver essentials to homes. For example, BeLoved Asheville, a local nonprofit, organized volunteers to bring gray water directly to residents of a public housing complex so that they could flush their toilets [27]. These efforts can be a model for similar distributions of essential resources as well as disseminating key information to residents with limited mobility or otherwise unable to leave their homes.

It is also necessary to consider the availability of medical care both immediately after a disaster as well as during the long-term recovery process. Residents who evacuated were challenged with securing medical care and caregivers in new locations. Those who remained in the area also experienced difficulty accessing medical services. Many respondents described difficulties with medical appointments, with closed offices and lack of cell and Internet service impeding access to Telehealth services. Securing necessary medications was also a challenge.

Medical providers might be encouraged to develop contingency plans that can ensure continuity of care in the event of a natural disaster. In addition, funds might be made available to providers that can support the general public after a disaster. The North Carolina Counseling Association [28], for instance, offered free mental health resources and counseling after Helene. Resources such as these may be particularly crucial for storm recovery, in acknowledging the mental health challenges that can arise or be exacerbated from a disaster.

Inclusive disaster relief is essential to address the needs of some of the most vulnerable residents in areas experiencing catastrophic events. Prior to mobilization, entities providing relief services can examine how their services can be more inclusive. And once on-site, efforts should be made to carefully examine and improve the accessibility of resources for people with a variety of disabilities. Results from this study shed light on barriers experienced by disabled residents as well as families with disabled members. Although the sample was limited and not representative of disabled residents as a whole, the respondents’ experiences can be generalized into specific recommendations for future disaster relief that is more inclusive for all residents of an area. It is believed that these recommendations can improve disaster relief for many types of events, beyond tropical storms and hurricanes, in different types of settings.
